# Fabrication and characterisation of magnetic graphene oxide incorporated Fe3O4@polyaniline for the removal of bisphenol A, t-octyl-phenol, and α-naphthol from water

**DOI:** 10.1038/s41598-017-11831-8

**Published:** 2017-09-12

**Authors:** Qingxiang Zhou, Yuqin Wang, Junping Xiao, Huili Fan

**Affiliations:** 1 College of Geosciences, China University of Petroleum Beijing, Beijing, 102249 China; 2 College of Chemistry and bioengineering, University of Science and Technology Beijing, Beijing, 100083 China

## Abstract

In this study, we fabricated a novel material composed of magnetic graphene oxide incorporated Fe_3_O_4_@polyaniline (Fe_3_O_4_@PANI-GO) using a modified Hummers’ method, solvothermal, and two-step polymerisation method. The resulting products were characterised by transmission electron microscopy (TEM), Fourier transform infrared spectroscopy (FT-IR), and X-ray diffraction (XRD). The results indicated that magnetic Fe_3_O_4_@PANI particles were successfully loaded onto the surface of the graphene oxide. Further Fe_3_O_4_@PANI-GO was investigated to remove bisphenol A(BPA), α-naphthol, and t-octyl-phenol (t-OP) from water samples by magnetic solid phase extraction. Under the optimal conditions, the Fe_3_O_4_@PANI-GO composite exhibited good adsorption capacity for t-OP, BPA, and α-naphthol, and the adsorption of these followed a pseudo-second-order kinetic model. Adsorption isotherms fit the Langmuir model, and the adsorption was an endothermic and spontaneous process. This work indicated that Fe_3_O_4_@PANI-GO earned great application prospect for removing phenolic contaminants from polluted water.

## Introduction

In recent years, magnetic nanoparticles (MNPs) have shown great technological significance in the areas of electronics, catalysis, therapy diagnosis, biosensors, and drug delivery due to their unique superparamagnetic properties^1^. Iron-based materials (e.g. Fe_3_O_4_) have drawn considerable attention as inorganic supports for the synthesis of organic-inorganic hybrid materials because of their potential application in information storage, drug delivery, targeting, and magnetic separation^2^. Fe_3_O_4_ nanoparticles possess many merits such as high surface area, inexpensiveness, easy separation by an external magnetic field, and high reusability, while they also are naturally hydrophilic due to the existence of plentiful hydroxyl groups on the particle surface^3^. However, since magnetite is highly susceptible to oxidation/dissolution, especially in acidic solutions^4, 5^, and is easily aggregated, all of these properties cause instability. Thus, magnetic adsorbents are difficult to directly use because of the aggregation and limited adsorption property. An effective strategy is to coat or modify iron oxide nanoparticles with other substrates to enhance the stability of the composite material and improve the special adsorption of target compounds, which is also a successful way to widen the application of the material by coating multifunctional groups on their surfaces.

Polyaniline (PANI) is one of the most technologically important materials based on its environmental stability in a conducting form, unique redox properties, and high conductivity with suitable dopants^6, 7^. The physicochemical properties of polyaniline and its potential applications in diverse fields such as battery, sensors, and wave-adsorption^8-10^ have been reviewed. PANI can be easily synthesised by either chemical or electrochemical methods. Recently, bifunctional Fe_3_O_4_@PANI nanocomposites have attracted intensive attention for application in nanomaterials due to their novel magnetic and conductive properties. Xuan *et al*. reported the synthesis of Fe_3_O_4_@polyaniline core-shell microspheres with well-defined blackberry-like morphology^6^. Zhao *et al*.^11^ prepared Fe_3_O_4_@PANI composite nanoparticles with a core-shell structure and measured their inductive heat property for localised hyperthermia. PANI was also used to easily and efficiently remove pollutants like heavy metal ions and organic contaminant from aqueous solutions^12–14^. In view of PANI polymers having a wealth of amino and benzene ring groups, the material can adsorb organic compounds and metal ions by π-π interaction and electrostatic interaction. Therefore, PANI is expected to be a promising adsorbent for the removal of aromatic compounds in water. However, the mechanical weakness and poor solubility of PANI greatly hinder further experimental investigation and commercial exploitation of the material^15^.

Graphene oxide (GO) consists of a hexagonal carbon network bearing hydroxyl and epoxide functional groups on its “basal” plane and is a single sheet of graphite oxide that exhibits good properties for many applications. It can be obtained by exfoliation of graphite oxide^16^ whereas the edges are mostly decorated by carboxyl and carbonyl groups^17^. These oxygen-containing functional groups can bind with metal ions and organic contaminants in water. Yang *et al*. found that the adsorption capacity of Cu(II) on GO was 10 times higher than that of Cu(II) on activated carbon^18^. Chang *et al*. prepared Fe_3_O_4_/graphene nanocomposites and achieved good adsorption performance for aniline and p-chloroaniline^19^. Xie *et al*. developed a facile chemical method to produce a superparamagnetic graphene oxide-Fe_3_O_4_ hybrid composite and which was successfully utilised for the removal of dyes from aqueous solution with high removal efficiency^20^. However, upon the removal of the hydrophilic functional groups on GO, it can lead to aggregated graphene sheets that are a few layers thick^21^. GO has high hydrophilicity and good dispersibility, making it suitable for direct application as an adsorbent for the separation/preconcentration of organic contaminants. Some graphene materials need to be centrifuged in the last separation steps in order to prepare graphene composite material with high specific surface area and stability^22^, which is significant for broadening the application of graphene oxide. There exist some reports on the application of PANI in the fabrication of a GO/PANI composite for supercapacitors and high-performance shielding materials^23–25^, etc., which suggests that PANI can be effectively anchored on a magnetic substrate via strong interactions with GO as the intermediate. This process not only reserves the oxygen-containing functional groups of GO, but also enhances the stability of the magnetic composite.

In present study, Fe_3_O_4_@PANI-GO composite was synthesised by decorating GO and PANI, which provided nitrogen-containing functional groups and protected the Fe_3_O_4_ nanoparticles. The prepared magnetic composites were investigated as magnetic adsorbents to remove BPA, t-OP, and α-naphthol from aqueous solution. The adsorption parameters of Fe_3_O_4_@PANI-GO for the removal of the three phenols from aqueous solution were investigated. The adsorption kinetics, isotherms, and thermodynamic studies were also performed to demonstrate the mechanism of the composite material toward BPA, α-naphthol, and t-OP.

## Results

### Morphology and structure

Fe_3_O_4_@PANI-GO composite was prepared and characterised by Fourier transform infrared spectrometry (FT-IR, Nicolet Magana-IR 750) in the 400 to 4000 cm^−1^ region. The shape and size distribution of the Fe_3_O_4_ and Fe_3_O_4_@PANI-GO hybrids were characterised by transmission electron microscopy (TEM, JEM2010F microscope) and scanning electron microscopy (SEM, CAMBRIDGE S-360 microscope). Powder X-ray diffraction (XRD) was performed on a Bruker D8-advance X-ray diffractometer at 40 kV and 40 mA for monochromatised Cu Kα (λ = 1.5406 Å) radiation.

**Figure 1:**
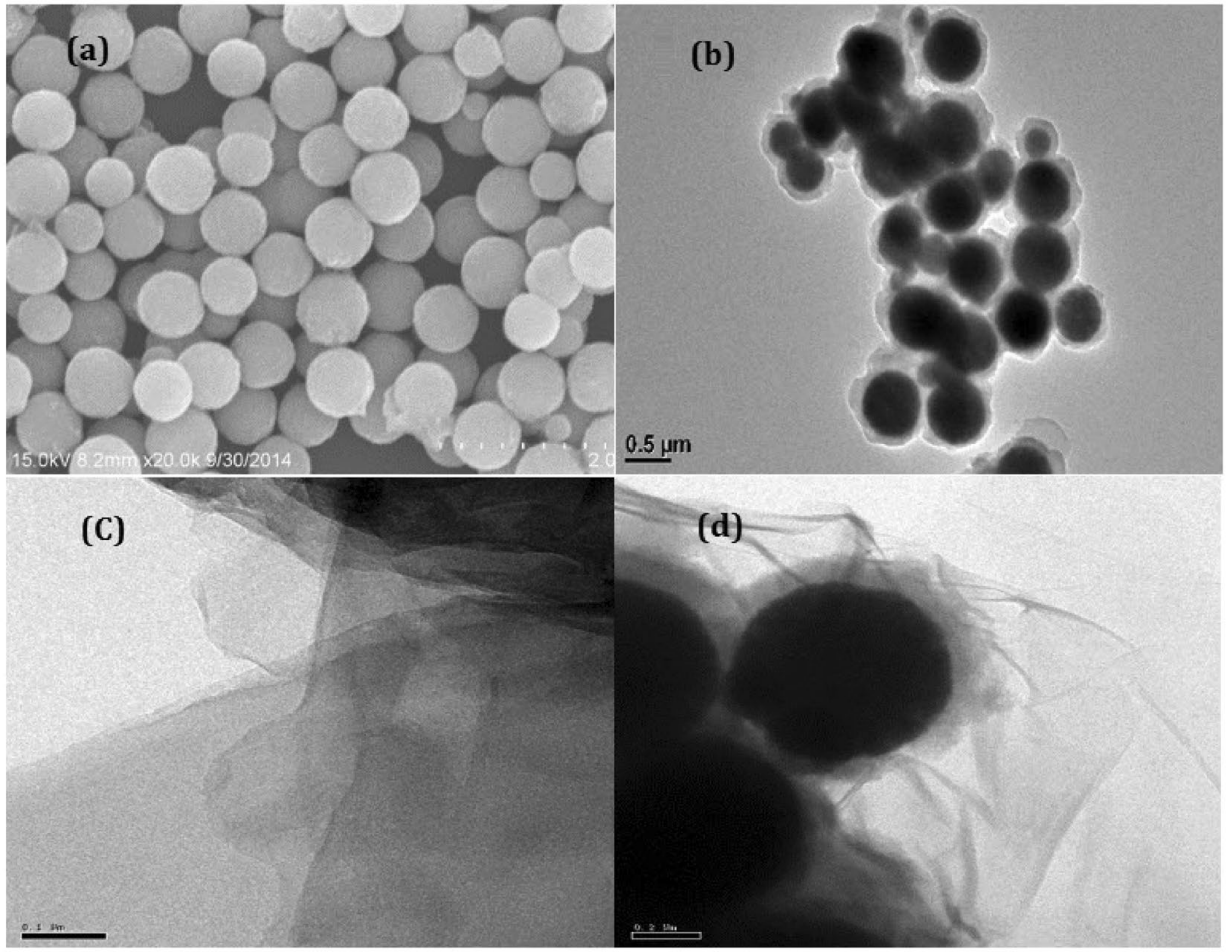
(**a**) SEM images of the as-prepared Fe_3_O_4_; (**b**) TEM images of Fe_3_O_4_@PANI core/shell composite, (**c**) GO, and (**d**) Fe_3_O_4_@PANI-GO.

Figure 1(a) shows the SEM of Fe_3_O_4_ nanoparticles, which have an average size of about 200–300 nm. Figure 1(b) displays the TEM images of the Fe_3_O_4_@PANI core-shell material in which a clear uniform shell of about 50 nm is observed, indicating the successful polymerisation of PANI on the surface of Fe_3_O_4_. Figure 1(c,d) shows the TEM images of original GO and Fe_3_O_4_@PANI-GO hybrids. It was also clear that Fe_3_O_4_@PANI particles highly covered the surface of GO nanosheets (Fig. 1(d)), indicating possible electrostatic attraction between graphene and Fe_3_O_4_@PANI microspheres.

**Figure 2:**
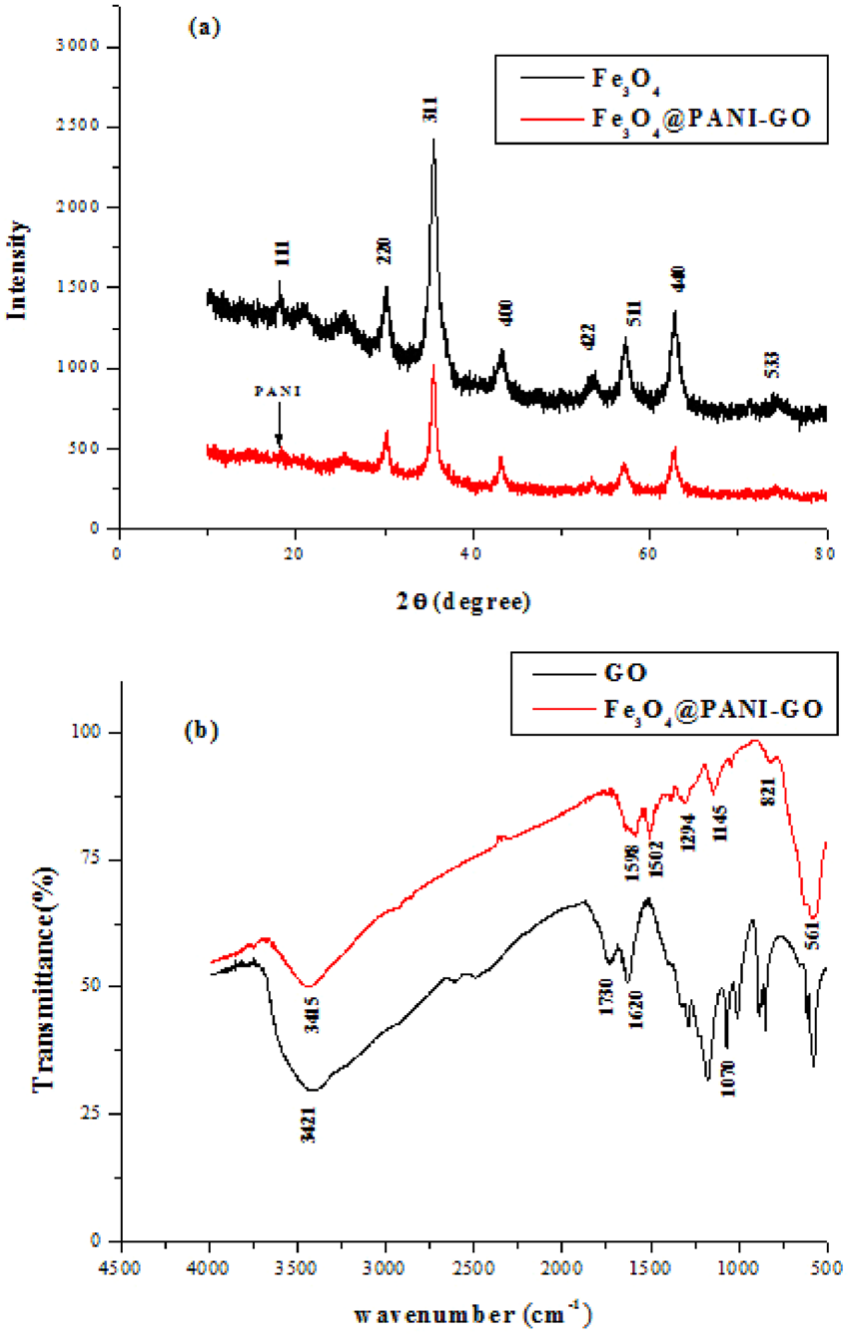
(**a**) XRD patterns of Fe_3_O_4_ and Fe_3_O_4_@PANI-GO; (**b**) FTIR spectra of pure GO and Fe_3_O_4_@PANI-GO composites.

The structure of Fe_3_O_4_@PANI on graphene oxide was corroborated by XRD measurements. As seen in Fig. 2(a), the XRD pattern of Fe_3_O_4_ and Fe_3_O_4_@PANI-GO exhibits two peaks; the main peaks of Fe_3_O_4_ nanoparticles at 2θ = 18.5°, 30.4°, 35.7°, 43.2°, 54.2°, 57.6°, and 63.1° are assigned to the (111), (220), (311), (400), (422), (511), and (440) reflections, respectively. The diffraction peaks of the graphene oxide composite material are consistent with Fe_3_O_4_ nanoparticles, indicating the presence of Fe_3_O_4_ nanoparticles in the composites. There are no obvious diffraction peaks for GO (002), suggesting that GO has good interaction with Fe_3_O_4_ and PANI, and PANI can be observed in the XRD of Fe_3_O_4_@PANI-GO at 2θ = 19.79° ^26^.

Figure 2b shows the FT-IR spectra of GO and Fe_3_O_4_@PANI-GO composite material. As GO is concerned, the bands at 1730 and 1070 cm^−1^ are assigned to the characteristic peaks of C=O and C–O–C, respectively. In the FT-IR spectra of Fe_3_O_4_@PANI-GO, the adsorption bands at 1598 and 1502 cm^−1^ are attributed to the stretching vibration of C=C/C–C of benzenoid ring and quinoid. The band at 1294 cm^−1^ is the stretching vibration of C–N, which is the characteristic spectral bands of PANI, while the in-plane bending vibration of C=H is at 1145 cm^−1^. As expected, the characteristic peaks of Fe_3_O_4_ microspheres appear around 561 cm^−1^ and are contributed to the Fe–O bond stretching^27–29^, and the broad and intense band at 3400 cm^−1^ is ascribed to the stretching of O–H. In comparison, the same of the peaks of Fe–O and O–H appeared in Fe_3_O_4_@PANI-GO composite material, which indicate that Fe_3_O_4_@PANI was successfully loaded onto graphene oxide.

### Optimization of adsorption

**Figure 3:**
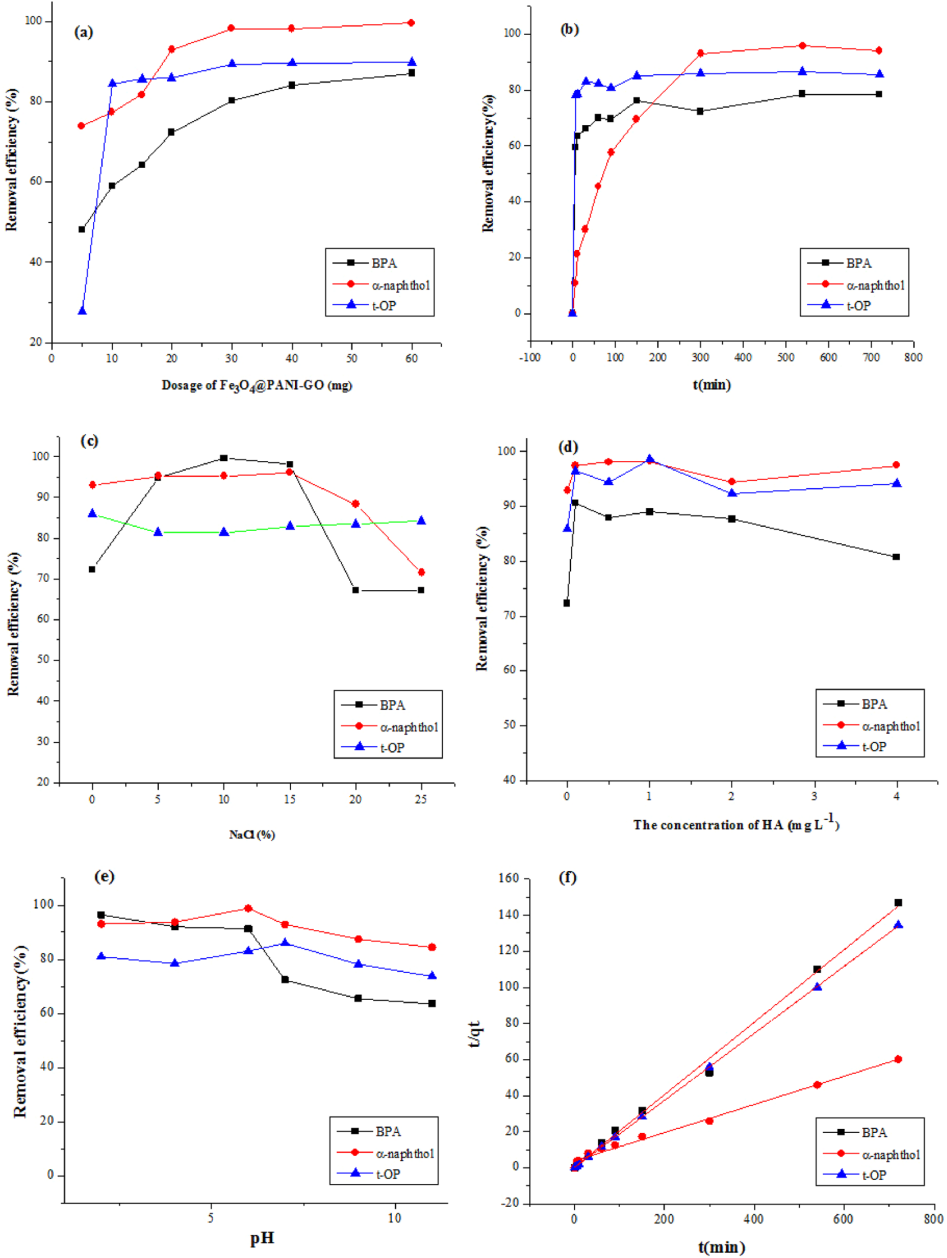
Optimisation of adsorption parameters and adsorption kinetics. Effects of the (**a**) amount of adsorbent, (**b**) contact time, (**c**) ionic strength, (**d**) concentration of HA, (**e**) pH, and (**f**) pseudo-second-order kinetics.

The important parameters that affect the adsorption such as amounts of adsorbents, sample pH, HA, ionic strength were optimized. The results showed that best results were obtained with 60 mg of Fe_3_O_4_@PANI-GO dosage at pH6. The salting-out effect and effect of HA were very small (See Fig. 3). The experimental data showed that the adsorption kinetics of BPA, t-OP, and α-naphthol conformed to pseudo-second-order kinetics and the data were exhibited in Fig. 3f and Table 1.

### Adsorption isotherm and thermodynamics

**Figure 4:**
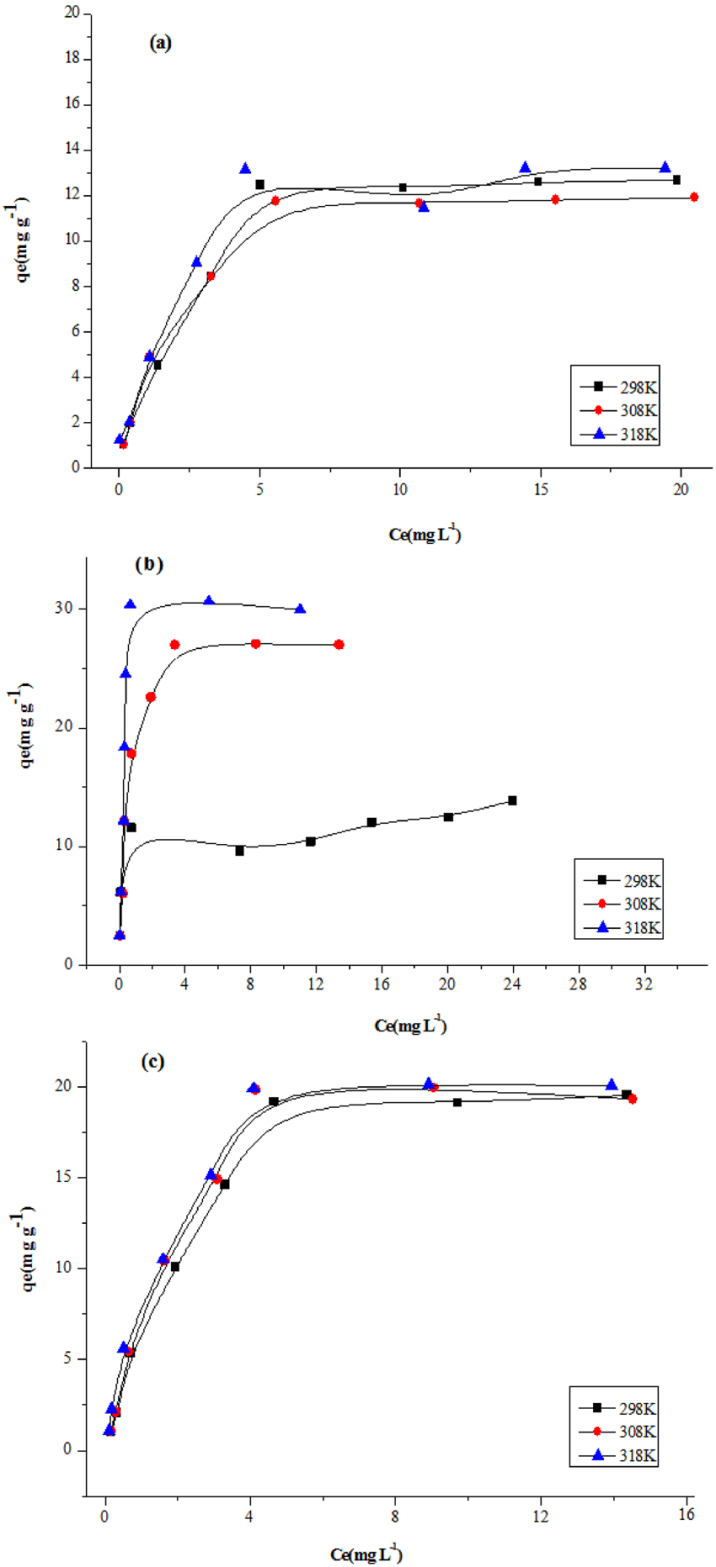
Adsorption isotherms of (**a**) BPA, (**b**) α-naphthol,and (**c**) t-OP.

The mechanism of adsorption is always in the center of our focusing, and several isotherm models were used to describe the adsorption behavior. The experimental results indicated that Langmuir model fit the adsorption data better than the Freundlich model for the adsorption of BPA, α-naphthol, and t-OP on the Fe_3_O_4_@PANI-GO magnetic composites. The thermodynamic data were calculated and demonstated that the adsorption was a spontaneous and endothermic process. These data were presented in Fig. 4, Tables 2 and 3.

**Table 2:** The parameters for isotherms at three different temperatures.

**T**	**phenols**	**Langmuir**				**Freundlich**			**Temkin**			**Dubinin–Radushkevich**		
		**R**_**2**_	**q**_**m**_ **(mg/g)**	**b(L/mg)**	**R**_**L**_	**R**_**2**_	**n**	**K**_**f**_ **(L/g)**	**R**_**2**_	**b**_**T**_	**K**_**T**_	**Kd**	**R**_**2**_	**E(KJ/mol)**
298 K	BPA	0.991	14.430	0.453	0.306	0.934	1.815	3.396	0.931	2.841	1.070	7.564	0.852	0.257
	α-naphthol	0.977	13.193	1.037	0.088	0.765	5.297	7.466	0.800	1.272	1.095	50.431	0.959	0.100
	T-OP	0.975	24.155	0.367	0.353	0.921	1.479	5.002	0.951	4.780	1.025	7.098	0.915	0.265
308 K	BPA	0.996	13.158	0.571	0.260	0.919	1.936	3.460	0.952	2.583	1.077	8.586	0.888	0.241
	α-naphthol	0.999	28.169	2.351	0.041	0.860	2.460	13.846	0.939	4.807	1.012	26.067	0.914	0.139
	T-OP	0.973	23.419	0.454	0.306	0.908	1.507	5.443	0.936	4.785	1.024	8.250	0.924	0.246
318 K	BPA	0.986	14.065	0.731	0.215	0.908	2.674	4.981	0.825	1.945	1.074	5.739	0.920	0.295
	α-naphthol	0.999	30.769	5.417	0.018	0.755	2.649	20.186	0.795	4.811	1.009	41.205	0.927	0.110
	T-OP	0.990	23.041	0.617	0.245	0.923	1.662	6.340	0.953	4.463	1.023	12.289	0.947	0.202

**Table 3:** Thermodynamic parameters for the absorption of phenols onto Fe_3_O_4_@PANI-GO.

Compound	T	−∆G^0^(kJ/mol)	∆S^0^(J/mol^.^K)	∆H^0^(kJ/mol)
BPA	298 K	2.367	101.41	27.69
	308 K	3.901		
	318 K	4.372		
α-naphthol	298 K	1.856	36.88	9.189
	308 K	1.985		
	318 K	2.519		
T-OP	298 K	1.599	76.17	21.25
	308 K	1.889		
	318 K	3.143		

### Reusability

**Figure 5:**
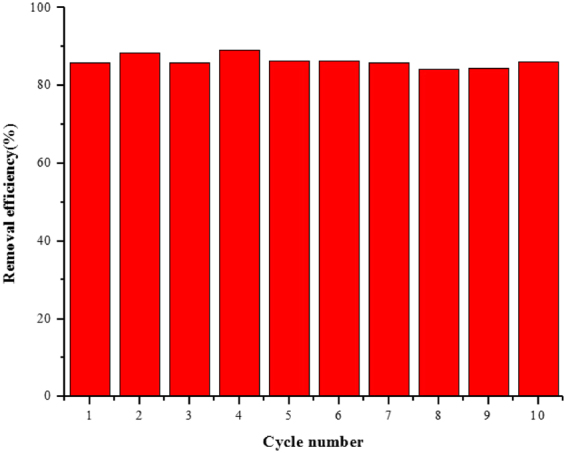
Recycling of Fe_3_O_4_@PANI-GO in the removal of t-OP at T = 298 K.

As a new adsorbent was concerned, the reusability was often an important parameter. In this study, it was investigated with ten recycles. The results were shown in Fig. 5, and the results indicated that the Fe_3_O_4_@PANI-GO magnetic composite was a good adsorbent with almost no loss of the recovery of BPA, α-naphthol, and t-OP after ten cycles.

## Discussion

### Adsorption

The adsorption of BPA, t-OP, and α-naphthol was performed by designing a series of experiments. The effect of the amount of adsorbent was investigated with an initial concentration of 5, 5 and 10 mg L^−1^ for BPA, t-OP and α-naphthol, respectively. (Fig. 3(a)). It was observed that the removal efficiency of the target compound adsorbed increased when the adsorbent dosage increased from 5 to 60 mg. The removal efficiency reached 91.32, 95.93, and 98.86% for BPA, t-OP, and α-naphthol, respectively. The removal rates of three phenols reached a steady state, and increased very small, and only the removal rate of BPA still had a slight increase, however the increase was very small. Therefore, 60 mg of Fe_3_O_4_@PANI-GO was used.

The effect of salinity was an important parameter and was often optimized parameter, herein it was checked with the concentration of NaCl in the range of 0–25% (w/v). Figure 3(c) shows the results of the effect of ionic strength on the adsorption of BPA, α-naphthol, and t-OP onto Fe3O4@PANI-GO. It was observed that the removal efficiency of BPA increased within the NaCl concentration of 0–10% (w/v) and then decreased to the initial level when the NaCl concentration up to 25%. As α-naphthol was concerned, its removal rate kept constant within NaCl concentration range of 0−15% and then decreased with increase of NaCl concentration up to 25%. For t-OP, no significant influence was observed in the concentration range of 5–25% (w/v).

The presence of humic acid (HA) may have affected the adsorption capacity of phenols due to its competition for surface adsorption sites on the composites. As shown in Fig. 3(d), an interesting phenomenon occurred in which a small amount of humic acid promoted the adsorption of three phenols. The best results were achieved when the concentration of humic acid was 0.1 mgL^−1^ for these three phenols. The adsorption efficiency of BPA increased to the maximum value of 90.7% with 0.1 mgL^−1^ humic acid, yet the continuous increase of humic acid concentration resulted in the decrease of BPA adsorption. For α-naphthol and t-OP, no significant influence of HA was observed in the HA concentration range of 0.1–4 mg L^−1^. The independence of humic acid concentration on phenols adsorption is important for the application of Fe_3_O_4_@PANI-GO in the removal of some organic pollutants from wastewaters since HA concentration may vary in different samples. Therefore, the effect of HA on the extraction efficiencies of target compounds in real water samples was negligible.

To optimise the pH for maximum adsorption capacity of BPA, α-naphthol, and t-OP on Fe_3_O_4_@PANI-GO magnetic composites, a series of adsorption experiments were carried at various pH values. The effects of pH on adsorption percentages of phenols were investigated from pH2 to pH11. As shown in Fig. 3(e), the Fe_3_O_4_@PANI-GO composite material adsorbed the phenols effectively in the range of pH 2–7. However, the adsorption rate declined sharply and even decreased to about 4.4% for α-naphthol at pH 9.0 and 11.0 and had no obvious effect for t-OP over the whole pH range. These phenomena could be explained by the net charge of graphene, BPA, and other organic matter at different pH values^30^. The strong adsorption of phenolic organic pollutants on the magnetic nanocomposites might be attributed to physical adsorption: the donor-acceptor interactions between the electrons of the aromatic ring and the graphene sheets^31^. Therefore, the kinetic and isotherm experiments were operated at pH 6.0.

### Adsorption kinetics

The effect of contact time on the amount of organics adsorbed was investigated, and results are presented in Fig. 3(b). It only took 5 min for BPA and t-OP to attain 59.41% and 78.04%, respectively, and for α-naphthol, the adsorption equilibrium was reached after 300 min. These results demonstrated that a fast adsorption process and the adsorbed amount of these phenols reached equilibrium values very quickly. The time-dependent adsorption capacity was obtained to study the kinetics for the adsorption of these phenols on Fe_3_O_4_@PANI-GO. The kinetics of adsorption is important considering that it controls the process efficiency. The adsorption model that describes the sorption of a solute onto a solid surface can be expressed by pseudo-first-order, pseudo-second-order, or intraparticle diffusion model^27–29^. The best-fit model was selected based on the linear regression correlation coefficient values (R_2_).

The pseudo-first-order kinetic model is expressed as:

1 $$\mathrm{ln}({q}_{e}-{q}_{t})=\,\mathrm{ln}\,{q}_{e}-{k}_{1}t$$

The pseudo-second-order kinetic model is present in the following equation as:

2 $$\frac{t}{{q}_{t}}=\frac{1}{{k}_{2}{{q}_{e}}^{2}}+\frac{t}{{q}_{e}}$$

The intraparticle diffusion model is defined as follows:

3 $${q}_{t}={k}_{i}{t}^{1/2}$$

where q_e_ and q_t_ were the amounts of Fe_3_O_4_@PANI-GO adsorbed (mg g^−1^) at equilibrium and at time t(min) respectively; and k_1_, k_2_, and k_i_ are the rate constants.

A good linear relationship between t/qt and t was obtained. The slopes and intercepts of each linear plot in Fig. 3(f) are used to calculate the kinetic parameters for BPA, t-OP, and α-naphthol adsorption, and the results are listed in Table 1. The correlation coefficients, R_2_, of the pseudo-second-order kinetic model for the adsorption of BPA, α-naphthol, and t-OP onto Fe_3_O_4_@PANI-GO were determined to be 0.9991, 0.9958, and 0.9999, respectively, which are much higher than that of the pseudo-first-order and intraparticle diffusion models. Clearly, the pseudo-second-order kinetic curves gave a good fit to the experimental kinetic data with a much higher correlation coefficient (R_2_). Furthermore, the experimental adsorption capacity (q_e_, exp) was also in accordance with the calculated adsorption capacity (q_e_, cal) obtained from the pseudo-second-order model. These results suggest that the pseudo-second-order kinetic model offers a more appropriate description of the adsorption process.

**Table 1:** Comparison results among the kinetic models for BPA, α-naphthol, and t-OP adsorption on Fe3O4@PANI-GO.

Compound	pseudo-first-order kinetic models		pseudo-second-order kinetic models		intraparticle diffusion model		
	R2	k1	R2	k2	R2	Ki	intercept
BPA	0.9245	0.0132	0.9991	0.0278	0.704	−5.1781	4.6182
α-naphthol	0.9333	0.0079	0.9958	0.0013	0.6097	−47.684	9.1623
T-OP	0.7047	0.0146	0.9999	0.098	0.648	−2.4027	5.2762

### Adsorption isotherms

Adsorption isotherms describe the distribution of adsorbed molecules between the liquid phase and solid phase. The adsorption isotherms for the removal of BPA, t-OP, and α-naphthol were studied using an adsorbent dosage of 20–50 mg. Langmuir, Freundlich, Temkin, and Dubinin-Radushkevich isotherm models were used to describe the adsorption process^32–35^.

The adsorption isotherm of the Langmuir model assumes monolayer adsorption on a perfectly smooth and homogeneous surface. It has been successfully applied to many pollutant adsorption processes from aqueous solution. The equation is expressed as:

4 $${q}_{e}=\frac{{q}_{m}b{c}_{e}}{1+b{c}_{e}}{R}_{L}=\frac{1}{b+{C}_{0}}$$

where q_e_ is the adsorption capacity (mg g^−1^) at the equilibrium point; C_e_ is the equilibrium concentration of BPA, α-naphthol, and t-OP (mg L^−1^); q_m_ represents the maximum adsorption capacity of the adsorbent (mg g^−1^); and b is the Langmuir adsorption constant (L mg^−1^).The value of R_L_ indicates the shape of the isotherm to be unfavourable (R_L_ > 1), linear (R_L_ = 1), favourable (0 < R_L_ < 1), or irreversible (R_L_ = 0) in which R_L_ values between 0 and 1 indicate favourable adsorption. Figure 4 exhibits the Langmuir adsorption isotherms of BPA, α-naphthol, and t-OP on as-prepared Fe_3_O_4_@PANI-GO at three different temperatures. The adsorption capacity, q_m_, and adsorption constant, b, can be determined from the slope and intercept of a linearized plot of C_e_/q_e_ vs C_e_, as presented in Table 2. The maximum adsorption capacities (q_m_) calculated according to the Langmuir model were 14.43, 13.19, and 24.15 mg g^−1^ for BPA, α-naphthol, and t-OP on Fe_3_O_4_@PANI-GO composites, respectively. The R_L_ values were obtained in the range of 0.0350–0.5638, thereby confirming that the adsorption is a favourable process. Comparing of the q_m_ values for phenols, the graphene oxide composite has a higher absorbability for t-OP than does BPA and α-naphthol.

The Freundlich isotherm is an empirical equation employed for heterogeneous systems and adsorption at multilayers. The equation is expressed as:

5 $${q}_{e}={K}_{f}{{C}_{e}}^{1/n}$$

where k_f_ and n are Freundlich constants that indicate the relative sorption capacity and sorption intensity, respectively. If 1 < n < 10, the adsorption is favourable^31^. Hence, it can be seen (Table 2) that the adsorption of BPA, α-naphthol, and t-OP on Fe_3_O_4_@PANI-GO composites were favourable in this research.

The Temkin isotherm considers the effects of indirect adsorbent/adsorbate interactions on adsorption isotherms. The isotherm assumes that the heat of adsorption of all the molecules in the layer decreases linearly with coverage due to adsorbent-adsorbate interactions. The equation is expressed as:

6 $${q}_{e}={b}_{T}\,\mathrm{ln}({K}_{T}{C}_{e})$$

Which can be linearized to:

7 $${q}_{e}={b}_{T}\,\mathrm{ln}\,{K}_{T}+{b}_{T}\,\mathrm{ln}\,{C}_{e}$$

where K_T_ is the constant of Temkin isotherm (g^−1^); and b_T_ is the Temkin isotherm constant related to the heat of adsorption (kJ mol^−1^).

The Dubinin-Radushkevich isotherm is used to estimate the characteristic porosity and the apparent free energy of adsorption. The Dubinin-Radushkevich equation is expressed as:

8 $${q}_{e}={q}_{m}\exp (-{K}_{D}{\varepsilon }^{2})$$

where K_D_ (mol^2^ kJ^−2^) is the mean free energy E (kJ mol^−1^) of adsorption per molecule of the sorbate when it is transferred to the surface of the solid from infinity in the solution, which was calculated by E = −(2K_D_)^−0.5^; and ε is Polanyi potential constant given as RT ln(1 + 1/C_e_). The E value can be used to estimate the type of adsorption. If 8 < E < 16 kJ mol^−1^, the adsorption can be explained by ion exchange. If E < 8 kJ mol^−1^, it is physical absorption, and if E > 16 kJ mol^−1^, it is chemical adsorption.

In Table 2, the higher correlation coefficients(R_2_) indicate that the Langmuir model fit the adsorption data better than the Freundlich model for the adsorption of BPA, α-naphthol, and t-OP on the Fe_3_O_4_@PANI-GO magnetic composites. The mechanism of Fe_3_O_4_@PANI-GO adsorption toward the phenols might be based on van der Waals interactions occurring between the hexagonally arrayed carbon atoms in the graphene oxide sheet and the aromatic backbones of the organics. The second reason might be due to the strong π-stacking interaction between the benzene ring of the organics and the large delocalized π-electron system of GO^32^. Thus, it is suggested that the graphene oxide magnetic adsorbent has a higher adsorption capacity for BPA, α-naphthol, and t-OP removal. Further, it is clear that the sorption energy values (E) of the Dubinin-Radushkevich model for BPA, α-naphthol, and t-OP are similar to each other, which are 0.257, 0.010, and 0.265 kJ mol^−1^, respectively, at 298k.These indicated that the adsorption was physical adsorption, and the positive values indicated that the adsorption process was endothermic.

### Thermodynamic studies

Batch adsorption was performed at different temperatures (298, 308, and 318 K), and the results are summarised in Table 3. The thermodynamic parameters were estimated in order to evaluate the feasibility and exothermic nature of the adsorption process. Free energy of adsorption (∆G°), enthalpy (∆H°), and entropy (∆S°) changes were calculated to predict the nature of adsorption.

The free energy of adsorption (∆G°) can be related to the equilibrium constant, K_0_ (L mol^−1^), where K_0_ can be obtained from the intercept of the plot ln(q_e_/C_e_) vs. q_e_. The adsorption standard free energy changes (∆G°) can be calculated according to following equation:

9 $${\rm{\Delta }}{G}^{0}=-RT\,\mathrm{ln}\,{K}_{0}$$

where R is the gas universal constant (8.314 J/mol K); K_0_ is the equilibrium constant; and T is the absolute temperature.

The van’t Hoff equation was used to determine K_0_:

10 $$\mathrm{ln}\,{K}_{0}=\frac{{{\rm{\Delta }}{\rm{S}}}^{0}}{2.303{\rm{R}}}-\frac{{{\rm{\Delta }}{\rm{H}}}^{0}}{2.303{\rm{RT}}}$$

Which can be converted to

11 $$\mathrm{ln}\,{K}_{0}=\frac{{{\rm{\Delta }}{\rm{S}}}^{0}}{{\rm{R}}}-\frac{{{\rm{\Delta }}{\rm{H}}}^{0}}{{\rm{RT}}}$$

12 $${{\rm{\Delta }}H}^{0}={{\rm{\Delta }}{\rm{G}}}^{0}+T{{\rm{\Delta }}{\rm{S}}}^{0}$$

where ∆H^0^ and ∆S^0^ values can be obtained from the slope and intercept by plotting lnK_0_ vs. 1/T. The thermodynamic parameters are listed in Table 3. The negative values of free energy of adsorption (∆G^0^) increased when the temperature increased, which indicates that the adsorption process is spontaneous. The positive standard enthalpy change (∆H^0^) also suggests that the interaction of BPA, α-naphthol, and t-OP adsorbed by Fe_3_O_4_@PANI-GO is endothermic, which is supported by the increased adsorption of BPA, α-naphthol, and t-OP with the increase in temperature.

### Regeneration

To investigate the possibility of regeneration of the Fe_3_O_4_@PANI-GO adsorbent, desorption experiments were performed in which organic pollutants were easily dissolved in organic solvents. Because of the theory of “similarity and intermiscibility”, we selected methanol as the desorption solvent. After adsorption, desorption was carried out by shaking the Fe_3_O_4_@PANI-GO with 3 mL methanol. 5 min was allotted as the extraction and desorption time, and the removal efficiencies of phenols are shown in Fig. 5.

From Fig. 5, it is observed that desorption of t-OP was achieved from the solution by using 3 mL methanol, and desorption ratio of 86.03% was obtained over ten adsorption/desorption cycles, which indicated that Fe_3_O_4_@PANI-GO sorbents were stable during the MSPE procedure and shows good reusability.

## Conclusions

In summary, we developed a facile method for the preparation of the magnetic composite material, Fe_3_O_4_@polyaniline with incorporated GO, and TEM, SEM, FT-IR, and XRD investigation revealed the characteristics of the composites. The Langmuir, Freundlich, Temkin, and Dubinin-Radushkevich adsorption models were applied to describe the equilibrium isotherms. The calculated maximum adsorption capacities were 14.43, 13.19, and 24.15 mg g^−1^ for BPA, α-naphthol, and t-OP on the Fe_3_O_4_@PANI-GO composite, respectively. In addition, the adsorption capacity of Fe_3_O_4_@PANI-GO for t-OP was higher. The negative adsorption standard free energy changes and positive standard enthalpy change indicate that the adsorption was spontaneous and endothermic. Furthermore, owing to the excellent dispersion in water and hydrophilicity of GO, the stability of the hybrid material in water is attributed to GO. Furthermore, the magnetic separation technology provided a rapid and effective method for separating magnetic materials from aqueous phase and displayed good regeneration capacity. This research indicates that Fe_3_O_4_@PANI-GO can be used as an effective sorbent for the simple and rapid removal of organic pollutants from water samples.

## Materials and Methods

### Chemicals and materials

Graphite powder (500 meshes) was purchased from J&K Chemical Ltd (Beijing, China). Concentrated sulfuric acid (H_2_SO_4_), K_2_S_2_O_8_, H_2_O_2_, KMnO_4_, HCl, Ferric chloride hexahydrate (FeCl_3_·6H_2_O), sodium acetate (CH_3_COONa), ethylene glycol (C_2_H_6_O_2_), ethanol, and ammonium peroxodisulfate ((NH_4_)_2_S_2_O_8_; APS) were all of analytical grade and used without further purification. Ultrapure water and double deionized water were used throughout the experiment.

### Preparation of graphene oxide (GO) and Fe_3_O_4_ nanoparticles

Graphene oxide (GO) was synthesised from natural graphite powder using a modified Hummers’ method^36, 37^. Fe_3_O_4_ microspheres were synthesised following a solvothermal method reported previously^38, 39^. FeCl_3_^.^ 6H_2_O (4.32 g) and sodium acetate (12.0 g) were dispersed in 80 mL ethylene glycol and stirred vigorously for 30 min at room temperature, then transferred to a Teflon-lined stainless steel autoclave (100 mL, capacity) and heated to reflux for 8 h at 200 °C. In this case, FeCl_3_·6H_2_O was used as the iron source, and NaAc was used as the reductant. NaAc was also used for electrostatic stabilisation to prevent the agglomeration of the particles and to assist in the reduction of Fe^3+^ to Fe_3_O_4_^40^. The obtained Fe_3_O_4_ was washed with deionized water and ethanol several times and then dried at 60 °C for 6 h.

### Preparation of Fe_3_O_4_@PANI-GO composite material

0.1 g Fe_3_O_4_ was dissolved in 50 mL deionized water containing 1 mL 0.02 M HCl aqueous solution in which HCl was used as a dopant. Then, FeCl_3_.6H_2_O and aniline monomer were added and sonicated for 10 min. After that, the mixture was mechanically stirred for 10 h in an ice bath for short-chain polymerization. APS and GO were then slowly added to the suspension under constant stirring. The long-chain oxidative polymerization was further continued for 12 h. After the reaction, the prepared particles were collected using a magnet and washed with deionized water and ethanol, then the obtained Fe_3_O_4_@PANI-GO composite was dried at 60 °C in a vacuum.

### Adsorption experiment

Adsorptions of BPA, t-OP, and α-naphthol by Fe_3_O_4_@PANI-GO magnetic composites were performed by batch adsorption techniques in glass vials at T = 25 ± 1 °C, except for the thermodynamic experiments in which additional temperatures of 35 ± 1 °C and 45 ± 1 °C were used. 25 mL solution spiked with a known initial concentration was shaken with 20 mg magnetic Fe_3_O_4_@PANI-GO on a shaker at 270 rpm.

The effects of pH, ionic strength, and humic acid and the adsorption kinetics and adsorption isotherms were investigated by batch experiments. The initial concentrations of the solutions were 5 mg L^−1^ for BPA and t-OP, and 10 mg L^−1^ for α-naphthol in the pH and kinetics experiments. The maximum adsorption capacity and adsorption isotherms were calculated according to different concentrations of organics in the range of 2–35 for α-naphthol and 1–30 mg L^−1^ for BPA and t-OP, respectively.

After adsorption, the solution was separated by a magnet and analysed using a high-performance liquid chromatography (HPLC, Shimadzu SPD-10A, Wondasil-C18, superb 5 µm) system equipped with a UV-Vis detector. The detection wavelength was set at 225, 225, and 280 nm for BPA, t-OP, and α-naphthol, respectively. The mobile phase was composed of methanol and water (80/20, v/v), and the flow rate was set at 1 mL min^−1^. The absorption capacity (q, mg g^−1^) was calculated using Equation (1):

13 $$q=\frac{({c}_{0}-{c}_{e})v}{m}$$

where C_0_ and C_e_ are the initial and equilibrium concentrations of phenols in the solution (mg L^−1^), respectively; m is the mass of the adsorbent (g); and V (L) is the solution volume.

### Data Availability

All data generated or analysed during this study are included in this published article.
